# Detecting impaired myocardial relaxation in sepsis with a novel tissue Doppler parameter (septal e′/s′)

**DOI:** 10.1186/s13054-017-1727-9

**Published:** 2017-07-14

**Authors:** David J. Clancy, Michel Slama, Stephen Huang, Timothy Scully, Anthony S. McLean, Sam R. Orde

**Affiliations:** Intensive Care Unit, Nepean Hospital, Kingswood, 2747 NSW Australia

**Keywords:** Sepsis, Diastolic function, Myocardial relaxation

## Abstract

**Background:**

Left ventricular diastolic dysfunction is associated with mortality outcomes in severe sepsis and septic shock. There are ongoing issues with diagnosing diastolic dysfunction in this cohort, partly owing to the poor applicability of traditional parameters in the hyperdynamic circulation. In this feasibility study, we sought to assess the utility of a novel parameter (septal e′/s′) to identify diastolic dysfunction in patients with severe sepsis and septic shock who had normal systolic function against the 2016 American Society Echocardiography and European Association of Cardiovascular Imaging (ASE/EACI) guidelines on diastolic dysfunction.

**Methods:**

In this prospective observational pilot study, patients identified as having severe sepsis and septic shock underwent transthoracic echocardiography on day 1 and day 3 of their intensive care unit admission. In patients with normal systolic function, septal e′/s′ was calculated using the peak modal velocity of the s′ compared with the e′ from the septal annulus tissue Doppler imaging and compared with their diastolic grade according to the 2016 ASE/EACI guidelines on diastolic dysfunction.

**Results:**

On day 1 of admission, 44 of 62 patients with severe sepsis and septic shock had normal systolic function. There was a strong association of those with diastolic dysfunction having a reduced septal e′/s′ compared with patients with normal diastolic function (AUC 0.91). A similar relationship was seen with patients who had indeterminate diastolic dysfunction. On day 3, 37 patients had normal systolic function. Again, there was a strong association of those with diastolic dysfunction and a reduced septal e′/s′ (AUC 0.95).

**Conclusions:**

A reduction in septal e′/s′ may indicate diastolic dysfunction in patients with severe sepsis and septic shock who have normal systolic function. As opposed to limited traditional measures of diastolic dysfunction, it is applicable in those with hyperdynamic systolic function.

**Electronic supplementary material:**

The online version of this article (doi:10.1186/s13054-017-1727-9) contains supplementary material, which is available to authorized users.

## Background

Diastolic dysfunction in severe sepsis and septic shock has been suggested to be associated with increased mortality [1]. One of the major issues in research to date is a large variation in the definition of diastolic dysfunction used [2–5], largely owing to the lack of a gold standard in diagnosing diastolic dysfunction. The previous reference standard issued by the American Society of Echocardiography (ASE) [6] has been limited by several factors, such as the mandatory inclusion of left atrial size that is assumed to increase in response to raised left atrial pressure [4, 7]. This may not be the case in the acute situation. The most recent recommendations from the ASE and the European Association of Cardiovascular Imaging (ASE/EACI) published in 2016 [8] are more flexible in recognizing that not all parameters which reflect raised left atrial pressure (i.e., left atrial size) are abnormal in diastolic dysfunction. Further, they recognize that, given the relationship between systolic function and myocardial relaxation, patients with abnormal systolic function must have a degree of impaired diastolic function.

These recent recommendations were designed on the basis of outpatient populations, limiting their applicability to critically ill patients. Further, despite the improvements made in defining diastolic dysfunction, caveats remain with regard to traditional parameters that can make the recognition of impaired relaxation difficult in the intensive care unit (ICU) population, particularly in those with normal or hyperdynamic systolic function, where cut-off values are determined on the basis of non-stressed hearts. A more appropriate measure might possibly reference the myocardial relaxation relative to the systolic function, based on theories that there is a link between systolic and diastolic function such as that due to myocardial fibre orientation [9–11].

In this pilot study, we investigated the feasibility of using a novel method to assess diastolic function in patients with severe sepsis and septic shock: the ratio of early myocardial relaxation versus systolic motion of the septal annulus with tissue Doppler imaging (the e′/s′ ratio) in those with normal systolic function. Our hypothesis is that the septal e′/s′ ratio would be reduced (i.e. septal annulus systolic motion exceeds that of diastolic motion) in those with normal ejection fraction (EF) and diastolic dysfunction (according to the 2016 ASE/EACI guidelines), and that this may be an indicator of relative impaired myocardial relaxation and might potentially present prior to traditional measures of diastolic dysfunction that are used as a surrogate of raised left atrial pressure. We also assessed the impact of fusion of the passive and active mitral inflow velocities on diastolic dysfunction. Fusion is classically associated with tachycardia and first-degree heart block, but it is not known to indicate diastolic dysfunction [12].

## Methods

We conducted a prospective, observational pilot study at the Nepean Hospital ICU, Sydney, Australia, from September 2014 to February 2016. The study was approved by the Nepean Blue Mountains Local Health District Research Governance Office (14/35-LR/14/Nepean/70). Because echocardiography is a standard procedure in critically ill patients in our unit, the need for consent was waived. Inclusion criteria were adult patients (>18 years old) admitted to the Nepean Hospital ICU with severe sepsis or septic shock. The definitions of severe sepsis and septic shock were based on the standard definitions at the time of enrolment of patients, rather than based on the subsequent Sepsis-3 definition [13]. Hence, severe sepsis was defined as having documented or a strong suspicion of infection, with at least two of four clinical signs of inflammation (body temperature >38 °C or <36 °C, heart rate >90 beats/minute, white blood cell count <4 × 10^9^/L or >12 × 10^9^/L, respiratory rate >20 breaths/minute or partial pressure of carbon dioxide <32 mmHg) with additional evidence of organ dysfunction. Septic shock was defined as sepsis with refractory hypotension requiring vasoactive treatment [14]. Exclusion criteria included pregnancy, congenital heart disease, artificial valve prosthesis, severe mitral pathology and inadequate image quality. Enrolment was done if trained sonographers or co-author SRO was available to complete studies on days 1 and 3.

Patient data collected included demographic and physiological data, Sepsis-related Organ Failure Assessment (SOFA) scores, fluid balance, inotropic use and mechanical ventilation parameters. Previous echocardiography reports (including diastolic dysfunction) were acquired when available, although the grading of diastolic dysfunction for these studies was not based on the 2016 ASE/EACI guidelines. Fluid balance was recorded using the electronic records at the time of initial and subsequent echocardiography to the nearest hour. The correlating input and output charts were also checked to ensure accuracy. SOFA scores were retrospectively calculated at the time of the echocardiographic studies. Current rates of noradrenaline infusion and total volume of noradrenaline infused were also recorded to the nearest hour.

### Echocardiography

Baseline comprehensive echocardiography was performed by certified sonographers or co-author SRO (an intensive care and echocardiography specialist) at the earliest time from admission (day 1). Parameters measured were in accordance with current practice and included left ventricular size, left ventricular EF, left atrial volume, mitral inflow velocity, septal and lateral annulus tissue Doppler, tricuspid regurgitation (TR) velocity and cardiac output. Measurements were averaged from three cardiac cycles if the patient was in sinus rhythm and from five cardiac cycles in those with atrial fibrillation. Tissue Doppler measurements were taken from the modal velocity (or peak intensity of the Doppler signal) rather than the peak of the waves, given the variable accuracy of peak tissue Doppler measurements in various machines [15]. A repeat study was performed as soon as feasible from day 3 of admission.

Normal systolic function was defined as an EF calculated by Simpson’s biplane method >51%. Hyperdynamic systolic function was considered present if the EF was >65%. Diastolic dysfunction was classified according to the 2016 ASE/EACI guidelines. As per the 2016 guidelines, if patients had normal left ventricular systolic function and no obvious structural heart problem, they were first screened for diastolic dysfunction via a separate algorithm before subsequent grading. Diastolic dysfunction was diagnosed if they fulfilled three of the following four criteria: increased left atrial volume, average E/e′ >14, septal e′ <7 cm/second *or* lateral e′ <10 cm/second, and/or a TR velocity >2.8 m/second. If only two of these conditions were met, patients were deemed to have indeterminate diastolic dysfunction. Where diastolic dysfunction was confirmed or if there was evidence of structural heart problems (i.e., wall hypertrophy or known ischaemic heart disease) patients were graded as mild (grade I), moderate (grade II) or severe (grade III). Mild dysfunction was deemed present if the mitral E peak velocity was <0.5 m/second and the ratio of early to late diastolic velocity of mitral inflow (E/A) was <0.8; severe dysfunction was deemed present if the E/A was >2; and moderate dysfunction was diagnosed if the E/A was within these two ranges and at least two of the following criteria were met: raised left atrial volume, TR velocity >2.8 m/second or average E/e′ >14. If less than one of the parameters was met, then left atrial pressure was considered not to be raised, and patients subsequently had grade I diastolic dysfunction. Further, if one of the parameters was missing, patients were deemed to have indeterminate diastolic dysfunction if only one of the remaining parameters was positive.

The e′/s′ ratio was calculated from measurements of the systolic and early diastolic filling velocity from the septal annulus tissue Doppler imaging, and it was considered to be reduced if it was <1. The ratio was also calculated using data from the lateral annulus; however, we felt that the e′/s′ from the septal annulus would be more accurate, given that there is less translational movement and less Doppler beam angle error [16]. Fusion of the mitral inflow velocity was described in patients whose atrial wave began when the E velocity was >0.2 m/second as grade 1 fusion. If the E and A waves were indistinguishable (or close to indistinguishable), they were denoted as grade 2 fusion.

### Data and statistical analyses

Statistical analysis was performed with JMP version 11 software (SAS Institute, Cary, NC, USA). Continuous variables are reported as mean ± SD or median ± IQR and were analysed between groups using analysis of variance. If a significant difference was found, between-group analysis was performed using Tukey’s honestly significant difference test. Categorical variables are expressed as number of patients and percent of group, with comparisons made by Pearson’s chi-square test or Fisher’s exact test if fewer than five patients were in a specific group. For unadjusted comparisons between groups, Student’s *t* test was used for normally distributed data, and the Wilcoxon signed-rank test was used for non-normally distributed data. ROCs were formed to assess sensitivity and specificity, and AUC was used to assess the ability of e′/s′ to distinguish the presence of diastolic dysfunction. Probability values are considered two-sided, and a *p* value <0.05 was considered significant. All echocardiograms were reviewed by two blinded examiners (TS, DJC). Inter-observer variability for e′ and s′ was tested with intra-class correlation coefficients (using two-way mixed model testing for absolute agreement using IBM SPSS Statistics version 24 software [IBM, Armonk, NY, USA]), and mean differences were tested using Bland-Altman plots. Grading of diastolic dysfunction was performed by two examiners (MS, DJC). Any discrepancies were resolved by consensus in the presence of an adjudicator (SRO).

## Results

**Fig. 1:**
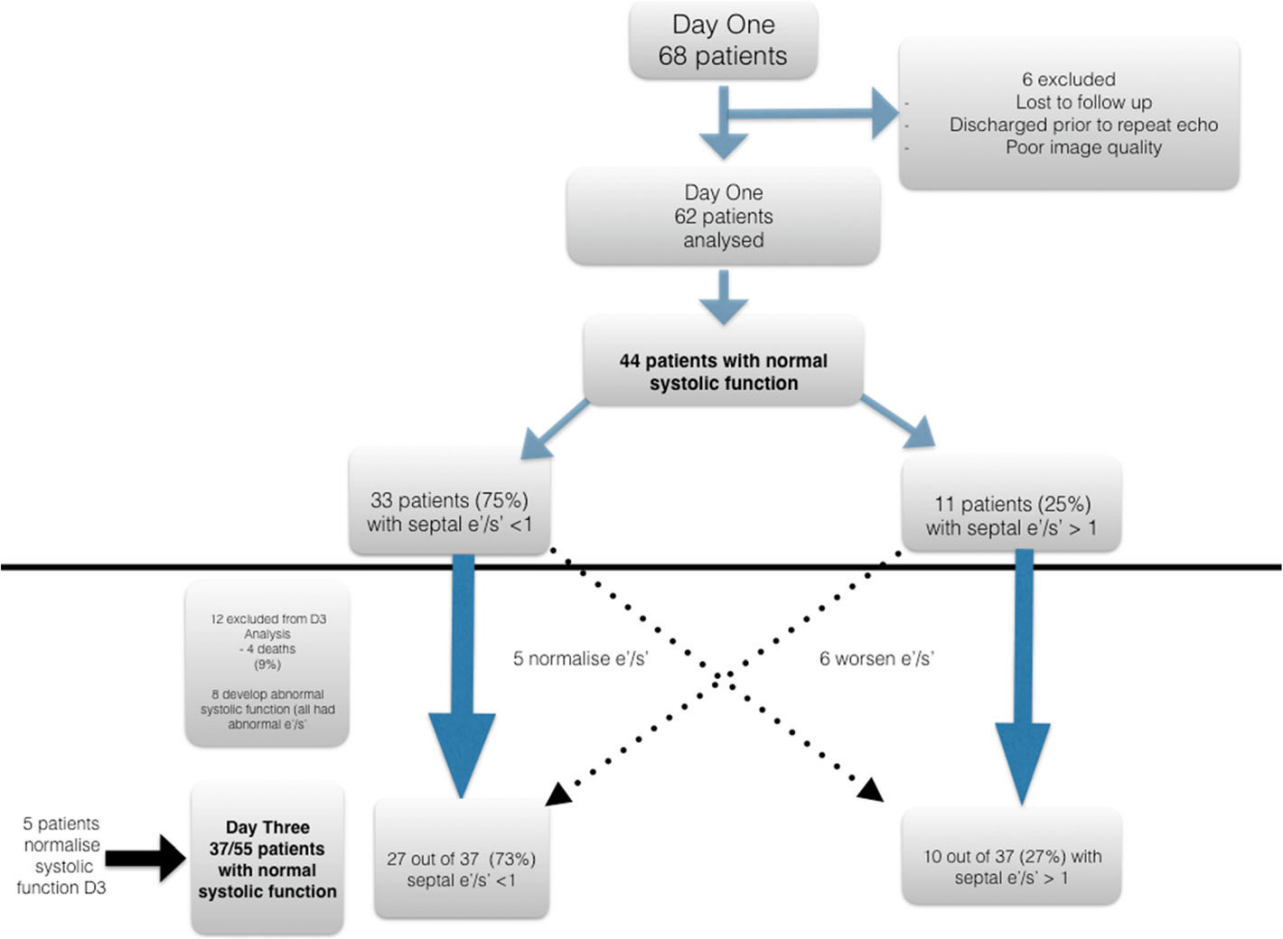
Flowchart of participants in study. *e′/s′* Ratio of early diastolic to systolic myocardial tissue velocity

Sixty-eight patients were included in the study. Six were lost to follow-up or had insufficient imaging and were excluded from analysis (*see* Fig. 1).

There were 44 patients on day 1 with normal systolic function; the remaining 18 patients had abnormal systolic function. On the basis of the 2016 ASE/EACI guidelines, 11 (25%) of the 44 patients with normal systolic function had normal diastolic function, with 20 (45%) having diastolic dysfunction and 13 (30%) whose diastolic dysfunction was unable to be determined. Intra-class correlation (Bland-Altman) between the reviewers for septal e′ on day 1 was 0.87 (95% CI 0.65–0.94) with a mean bias of 0.009 (95% CI 0.005–0.013). The correlation for septal s′ on day 1 was 0.9 (95% CI 0.82–0.95) with a mean bias of 0.004 (95% CI −0.0005 to 0.009).

**Fig. 2:**
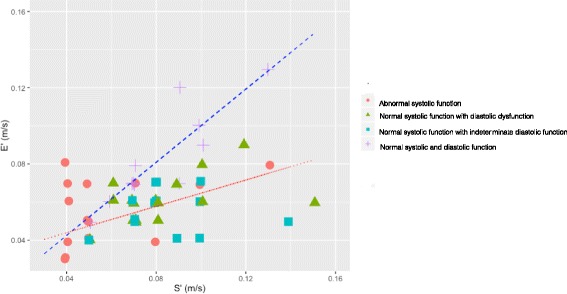
Septal e′ versus s′ in all patients on day 1. The *dashed blue line* represents the linear relationship between septal e′ and s′ for patients with normal systolic and diastolic function (e′ = 0.96 s′ + 0.004, *r* ^2^ = 0.0762, *p* < 0.001). The *red dotted line* represents the relationship between septal e′ and s′ for those with normal systolic function but abnormal diastolic function (e′ = 0.346 s′ + 0.0296, *r* ^2^ = 0.34, *p* = 0.007). Those with normal systolic function but indeterminate diastolic function did not have a significant linear relationship (e′ = 0.0782 s′ + 0.049, *r* ^2^ = 0.02, *p* = 0.628), but all patients had a septal e′/s′ ratio that was lower than the line of best fit for the normal systolic and normal diastolic function group. Those with abnormal systolic function had a much weaker linear relationship (e′ = 0.34 s′ + 0.03, *r* ^2^ = 0.24, *p* = 0.04). *e′/s′* Ratio of early diastolic to systolic myocardial tissue velocity

The relationship of e′ to s′ measured on the septal annulus in all patients on day 1 is detailed in Fig. 2. Those with normal systolic and diastolic function on day 1 had a significant strong linear relationship (*r* ^2^ = 0.762, *p* < 0.001). Those with abnormal systolic function had a much weaker linear relationship (*r* ^2^ = 0.24, *p* = 0.04). Of those with abnormal systolic function, 89% had evidence of impaired relaxation with either a reduced septal or lateral e′. The patients with normal systolic function and abnormal diastolic dysfunction had a reduced e′ relative to s′, but there was still a significant relationship seen (*r* ^2^ = 0.34, *p* = 0.007). Those whose diastolic function was indeterminate did not seem to have a significant relationship (*r* ^2^ = 0.02 *p* = 0.628); however, all patients had a septal e′/s′ ratio that was lower than the line of best fit for the normal systolic and normal diastolic function group.

**Fig. 3:**
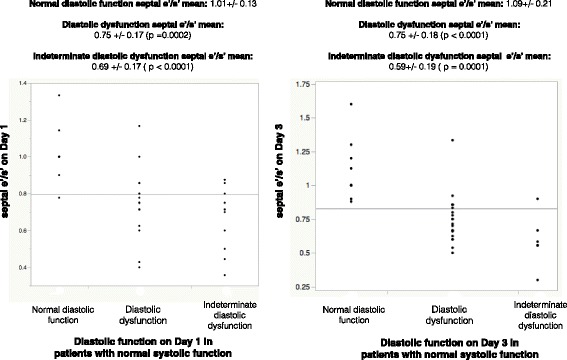
Patients with normal systolic function on day 1 and day 3 with presence of diastolic dysfunction versus septal ratio of early diastolic to systolic myocardial tissue velocity (e′/s′)

Baseline demographics for the patients with normal systolic function are included (Table 1). Of 44 patients, 33 had a septal e′/s′ <1, with the remaining 11 having an e′/s′ >1. There was no statistical difference between those with abnormal and normal septal e′/s′ regarding heart rate, SOFA score, positive end-expiratory pressure or fluid balance. Those with a reduced septal e′/s′ had a higher total use of noradrenaline on day 1 (Table 1). There was no difference between the two groups in cardiac output, stroke volume, or systolic tissue velocity at the septum (s′). There was no difference between the groups in regard to mean E/e′, left atrial volume or TR velocity. The group with a reduced septal e′/s′ had a higher incidence of septal hypertrophy and reduced e′. Although there was a significantly lower septal e′/s′ ratio in those with diastolic dysfunction (according to the 2016 ASE/EACI definition) than in those with normal diastolic function (see Fig. 3), there was no difference detected between the grades of diastolic dysfunction (Additional file 1). The septal e′/s′ was significantly reduced in those with indeterminate diastolic dysfunction compared with patients with normal diastolic dysfunction. This relationship was also displayed in the 12 patients (27%) with hyperdynamic systolic function, with the mean e′/s′ in those with normal diastolic function being 0.98 ± 0.04 compared with those with diastolic function (mean e′/s′ 0.65 ± 0.22, *p* = 0.03) and those with indeterminate function (mean e′/s′ 0.54 ± 0.17, *p* = 0.003). Only one of these patients had a reduced septal e′ (<7 cm/second). Of note, there was no difference in the lateral e′/s′ between those with normal diastolic function (mean 1.03 ± 0.22) and abnormal diastolic function (0.95 ± 0.3) or indeterminate diastolic function (0.89 ± 0.22)

**Table 1:** Baseline demographics of all patients, those with septal e′/s′ <1 and those with septal e′/s′ >1 on days 1 and 3

	Day 1			Day 3		
Variable	All patients, day 1 (*n* = 44)	Septal e′/s′ <1, *n* = 33 (75%)	Septal e′/s′ >1, *n* = 11 (25%)	All patients day 3 (*n* = 37)	Septal e′/s′ <1, *n* = 27 (73%)	Septal e′/s′ >1, *n* = 10 (27%)
Demographics						
Age, years	63 ± 12	65 ± 10	56 ± 15	62 ± 11	64 ± 12^a^	56 ± 9
Male sex	19 (43%)	13 (39%)	6 (55%)	17 (46%)	13 (48%)	4 (40%)
Mortality (ICU)	9 (20%)	7 (21%)	2 (18%)	5 (14%)	5 (19%)	0
Mortality (hospital)	12 (28%)	10 (30%)	2 (18%)	9 (24%)	9 (33%)	0
Past medical history						
IHD	10 (23%)	9 (27%)	1 (9%)	7 (19%)	7 (26%)	0
Diabetes mellitus	12 (28%)	11 (33%)	1 (9%)	10 (27%)	7 (26%)	3 (30%)
Hypertension	25 (57%)	21 (64%)	4 (36%)	22 (59%)	18 (67%)	4 (40%)
Known diastolic dysfunction	5 (11%)	4 (12%)	1 (9%)	3 (8%)	3 (11%)	0
Chronic kidney injury	8 (18%)	8 (24%)	0	8 (22%)	7 (4%)	1 (10%)
Clinical data						
Ventilator days	5 (1–9)	5 (2–9)	3 (0–6)	5 (1–8)	5 (1–9)	4 (2–8)
HR (on day of study)	96 ± 19	96 ± 17	96 ± 25	89 ± 18	89 ± 19	90 ± 15
Arrhythmia	8 (18%)	5 (15%)	3 (27%)	7 (19%)	7 (26%)	0
SOFA	9 (7–12)	9 (6–13)	10 (7–12)	6 (3–9)	6 (3–11)	6.5 (3–8)
PEEP, cmH_2_O	8 (5–10)	10 (7–14)	8 (5–10)	7 (5–10)	5 (7–10)	6 (5–11)
Fluid balance, ml	1042 (349–2391)	1174 (363–2425)	544 (332–2120)	2588 ± 3958	3012 ± 4203	3106 ± 1443
Noradrenaline, total mg	155 (45–334)	200^a^ (70–362)	49 (4–158)	441 (46–841)	364 (38– 851)	467 (94–829)

*Abbreviations: e′/s′* Ratio of early diastolic to systolic myocardial tissue velocity, *HR* Heart rate, *ICU* Intensive care unit, *IHD* Ischaemic heart disease, *PEEP* Peak end-expiratory pressure, *SOFA* Sepsis-related Organ Failure Assessment

^a^ *p* < 0.05

A further 12 patients were excluded from analysis on day 3: 4 died, and 8 patients developed subsequent systolic dysfunction. All of these latter eight patients had abnormal septal e′/s′ on day 1. Five patients who had abnormal systolic function on day 1 were included in the day 3 analysis because their systolic function had improved to normal. Of the 37 patients with normal systolic function on day 3, 27 had a reduced septal e′/s′ (<1), with the remaining 10 having an e′/s′ >1. The intra-class correlation coefficient for septal e′ was 0.9 (95% CI 0.73–0.96) with a mean bias of −0.0075 (95% CI −0.012 to −0.003), and for s′, the intra-class correlation coefficient was 0.86 (95% CI 0.74–0.93) with a mean bias of 0.0072 (95% CI −0.001 to 0.0.16). There was no difference between the two groups in terms of heart rate, SOFA score, fluid balance or noradrenaline use on day 3, although those with a reduced septal e′/s′ were older (Table 1). Those with reduced septal e′/s′ had a higher incidence of septal e′ <7 cm/second and higher mean E/e′, left atrial volume and TR velocity. Again, there was no difference between the groups in terms of cardiac output, stroke volume or septal s′ measured (Table 2).

**Table 2:** Echocardiography parameters of patients with septal e′/s′ <1 and patients with septal e′/s′ >1 on days 1 and 3

	Day 1		Day 3	
Echocardiography parameter	Septal e′/s′ <1 (*n* =33)	Septal e′/s′ >1 (*n* = 11)	Septal e′/s′ < 1 (*n* = 27)	Septal e′/s′ >1 (*n* =10)
Mean septal e′/s′	0.71 ± 0.14^a^	1.05 ± 0.11	0.70 ± 0.15^a^	1.16 ± 0.20
Septal hypertrophy, *n*	15^a^	1	12	1
E/e′ >14, *n*	10	1	12^a^	0
Mean E/e′	13 ± 5	10.6 ± 3.15	14.5 ± 8^a^	8.8 ± 2.6
Septal e′ <7 cm/s, *n*	25^a^	3	19^a^	1
Mean septal e′, cm/s	5.7 ± 1^a^	8 ± 3	6 ± 2^a^	9 ± 2
Lateral e′ <10 cm/second, *n*	28	8	18	4
Average lateral e′, cm/second	8 ± 2	8.6 ± 2	8 ± 3^a^	10 ± 2
Increased left atrial volume, *n*	21	5	22	4
Mean left atrial volume, ml	66 ± 27	59 ± 21	84 ± 33^a^	55 ± 22
TR velocity >2.8 m/second, *n*	9	2	10	1
TR velocity, m/second, average	2.6 ± 0.7	2.4 ± 0.6	2.8 ± 0.6^a^	2.2 ± 0.5
Mitral s′ average, cm/second	8 ± 2	7.5 ± 2	8 ± 2	7.6 ± 1
Cardiac output, L/minute	6.2 ± 2.2	5.4 ± 1.5	6.7 ± 2	6 ± 1.5
SV, ml	65 ± 24	61 ± 17	72 ± 21	66 ± 10
VTI, cm	20 ± 6.5	17 ± 4.4	21 ± 5	19 ± 2.3
Diastolic dysfunction	18^a^	2	19^a^	1
Indeterminate diastolic dysfunction	13^a^	0	6^a^	1

*Abbreviations: E/e′* Ratio of early diastolic mitral inflow velocity to early diastolic myocardial tissue velocity, *e′/s′* Ratio of early diastolic to systolic myocardial tissue velocity, *SV* Stroke volume, *TR* Tricuspid regurgitation, *VTI* Velocity time integral

^a^ *p* < 0.05

Of the 37 patients, 10 (27%) had normal diastolic function on day 3, 20 (54%) had diastolic dysfunction and 7 (19%) were indeterminate according to the 2016 guidelines. Those with gradable and indeterminate diastolic dysfunction again had a significant reduction in their septal e′/s′ (Fig. 3), with all grades of diastolic dysfunction having a lower mean septal e′/s′ than normal patients (Additional file 2). However, again, there was no difference in septal e′/s′ between the grades of diastolic dysfunction. The trend between groups was seen in those with hyperdynamic systolic function (*n* = 9 [24%]) on day 3: Those with normal diastolic function had a mean septal e′/s′ of 1.05 ± 0.2, patients with diastolic dysfunction had a mean septal e′/s′ of 0.67 ± 0.11, and the indeterminate patients had a mean septal e′/s′ of 0.68 ± 0.19 (not statistically significant). In contrast, there was no difference in lateral e′/s′ in those with normal diastolic function (mean 1.04 ± 0.21) and diastolic dysfunction (mean 0.94 ± 0.2).

**Fig. 4:**
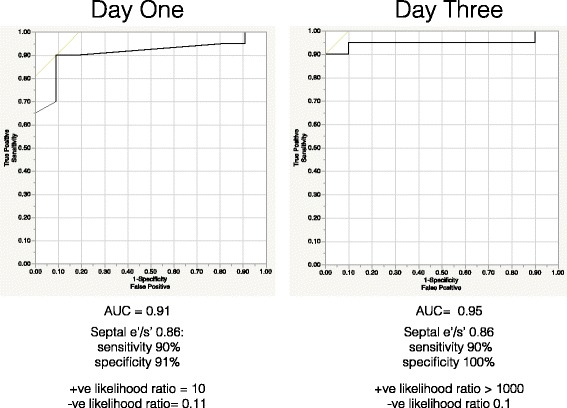
ROC curves for day 1 and day 3 septal ratio of early diastolic to systolic myocardial tissue velocity (e′/s′) versus diastolic dysfunction in patients with normal systolic function

The ROC curves for septal e′/s′ on both days are shown in Fig. 4 with AUCs of 0.91 on day 1 and 0.95 on day 3. An e′/s′ ratio of 0.86 on day 1 had a positive likelihood ratio of 10 for detecting diastolic dysfunction and a negative likelihood ratio of 0.11, and on day 3, the same septal e′/s′ ratio had a positive likelihood ratio >1000 and a negative likelihood ratio of 0.1.

On both days 1 and 3, in patients with normal EFs, increasing tachycardia was associated with increasing fusion of the mitral inflow velocity (Additional file 3). There was no relationship with fusion and diastolic dysfunction on either day.

## Discussion

Diastolic dysfunction remains difficult to diagnose, particularly in patients with sepsis and septic shock. Consensus expert recommendations are used as reference standards and are based largely on surrogate markers of raised left atrial pressure which have limited applicability outside the outpatient setting and have not been validated in the critically ill [6], particularly in conditions where filling pressures may be low, such as in sepsis. Caveats to echocardiographic markers of raised left atrial pressure (e.g., left atrial volume, E/A and E/e′) exist in the critically ill, including compliance of the left atrium and its ability to increase its volume in acute states, pre-load dependence [17], effects of positive pressure ventilation [18] on mitral inflow velocity and angle dependence of tissue Doppler [19]. Further, the parameters used are age-dependent. In the setting of systolic dysfunction, this is not as much an issue as myocardial relaxation, and diastolic function will be abnormal in this setting, as recognized in the recently published 2016 ASE/EACI guidelines on diastolic dysfunction and as demonstrated by the reduction in septal or lateral e′ in nearly 90% of those with systolic dysfunction. Issues may arise, however, when trying to assess the patient with normal systolic function.

In healthy hyperdynamic hearts (i.e. during exercise), both systolic function (estimated by the s′ wave on tissue Doppler) and the myocardial relaxation velocity (e′) increase with demand [20]. A similar relationship was seen in our hypothesis-generating series of patients with severe sepsis and septic shock if they had normal systolic and diastolic function (based on the current ASE/EACI guidelines) on day 1 (see Fig. 2). In the presence of normal systolic function, those with diastolic dysfunction have a reduced septal e′ relative to the s′ wave, reflecting possible abnormal myocardial relaxation comparable to the systolic function. Those with indeterminate diastolic dysfunction had a similar relationship. We note that this relationship was not seen on the lateral annulus, potentially because of the increased Doppler angle and translational movement seen when performing tissue Doppler imaging on the lateral annulus. Given that the septal e′/s′ was not significantly different in worsening grades of diastolic dysfunction, we do not propose that this is a surrogate measure of raised left atrial pressure; rather, it is an indicator of worsening intrinsic myocardial relaxation that is relatively load-independent (compared with E/A ratio and E/e′) and can still be used in the hyperdynamic circulation.

Although those with a reduced septal e′/s′ had no significant difference in regard to markers of raised left atrial pressure, such as E/e′, left atrial volume and tricuspid regurgitant velocity on day 1, by day 3, the mean of each parameter was increased. This is despite there being no difference between the two groups in factors that would affect pre-load, namely fluid balance and positive end-expiratory pressure. This suggests that markers of raised left atrial pressure are not exaggerated early and may take time to develop in the presence of decreased myocardial relaxation, supporting previously held views that markers of diastolic dysfunction such as left atrial volume are not as sensitive in acute states. To our knowledge, there are no specific data on how quickly left atrial volume increases as a consequence of raised left atrial pressure. This further raises the debate regarding defining diastolic dysfunction: Should the critical care physician be concerned primarily with measures denoting left atrial pressure as a surrogate of pulmonary capillary wedge pressure, which differs from left ventricular end-diastolic pressure, or should the focus be on detecting impaired myocardial relaxation [8]? Unfortunately, the term *diastolic dysfunction* may blur these different distinctions.

The presence of diastolic dysfunction in severe sepsis and septic shock has significant clinical implications. Several studies and a subsequent meta-analysis have indicated an increase in mortality in those patients with diastolic dysfunction [1]. One of the many hypotheses surrounding the improved outcomes in the use of beta-blockade and noradrenergic sparing agents (i.e. vasopressin) in severe sepsis is that lowering the heart rate may improve diastolic function [21–23]. This may be important because the proposed increased efficiency of diastolic filling in tachycardia (frequency-dependent acceleration of relaxation) is limited in sepsis [24]. One of the largest studies to date highlighted that left ventricular diastolic dysfunction (but not systolic function) had a significant correlation with raised troponins in severe sepsis, which is known to be a predictor of mortality [25]. This relationship of raised troponins and diastolic dysfunction may reflect impaired myocardial relaxation from myocardial oxygen supply demand imbalance, which may result from excessive catecholamines, tachycardia and/or microvascular dysfunction. This potential ischaemia resulting in diastolic dysfunction makes it imperative that myocardial work and oxygen demand be reduced.

We postulate that in normal and hyperdynamic hearts, the existence of an abnormally reduced septal e′/s′ may indicate impaired myocardial relaxation and potentially the need for rate control to improve diastolic filling time and reduce myocardial oxygen demand. Interestingly, all patients who developed systolic dysfunction between day 1 and day 3 had an abnormal septal e′/s′ on day 1. Whilst Weng et al. [26] found that an increased systolic myocardial velocity measured at the mitral annulus (>9 cm/second), indicating a hyperdynamic state, was associated with mortality in severe sepsis, we propose that it is those with abnormal relaxation in the setting of a hyperdynamic circulation who are increasingly at risk and would perhaps benefit from beta-blockade. In our sample, septal e′ was reduced in most patients with diastolic dysfunction. However, in the limited number of patients with a hyperdynamic circulation, the majority had a normal e′ even in the presence of impaired or indeterminate diastolic function.

Our study has several limitations. This is a single-centre study, limiting the number of patients recruited, and further, given the restraints of available sonographers, there were likely a significant number of patients missed in the study time period. A significant proportion of patients with indeterminate diastolic dysfunction based on the 2016 ASE/EACI guidelines on day 3 had missing data, which may have changed their grading. The 2016 ASE/EACI diastolic guidelines at best remain a standard reference measure for comparison against septal e′/s′ rather than a gold standard. Further, a high proportion of patients had increased myocardial wall thickness, indicating that they would likely have had diastolic dysfunction prior to their ICU presentation. Attempts to clarify pre-existing diastolic dysfunction by searching through patient history revealed limited documentation of pre-existing diastolic dysfunction. Although tissue Doppler parameters are relatively load-independent in relation to mitral inflow velocities, they have been demonstrated to change with large pre-load variations [17]. As such, septal e′/s′ potentially could vary at the extremes of volume states. Further prospective studies are warranted to address some of these limitations, including assessment of the effect of fluid balance on septal e′/s′.

## Conclusions

A reduction of septal e′ relative to s′ in patients with normal systolic function in the presence sepsis and septic shock may indicate impaired myocardial relaxation and confirm diastolic dysfunction. As opposed to limited traditional measures of diastolic dysfunction, it is applicable in those patients with hyperdynamic circulations.

## Additional files

Additional file 1: Patients with normal systolic function on day 1: septal e′/s′ versus diastolic grade. (PPTX 100 kb)

Additional file 2: Patients with normal systolic function on day 3: septal annulus e′/s′ versus diastolic grade. (PPTX 96 kb)

Additional file 3: Comparison of heart rate and grades of E/A fusion. (PPTX 184 kb)

## Abbreviations

A: Late diastole mitral inflow velocity
ASE/EACI: American Society of Echocardiography/European Association of Cardiovascular Imaging
E: Early diastole mitral inflow velocity
E/A: Ratio of early to late diastolic velocity of mitral inflow
E/e′: Ratio of early diastolic mitral inflow velocity to early diastolic myocardial tissue velocity
e′: Early diastolic myocardial tissue velocity
e′/s′: Ratio of early diastolic to systolic myocardial tissue velocity
EF: Ejection fraction
HR: Heart rate
ICU: Intensive care unit
IHD: Ischaemic heart disease
PEEP: Peak end-expiratory pressure
s′: Peak systolic myocardial velocity
SOFA: Sepsis-related Organ Failure Assessment
SV: Stroke volume
TR: Tricuspid regurgitation
VTI: Velocity time integral
