# Supplementary figures and images for: Detecting impaired myocardial relaxation in sepsis with a novel tissue Doppler parameter (septal e′/s′)
Source: Crit Care. 2017 Jul 14;21:175. doi: 10.1186/s13054-017-1727-9 (PMC5512826; doi:10.1186/s13054-017-1727-9)

## Slide 1
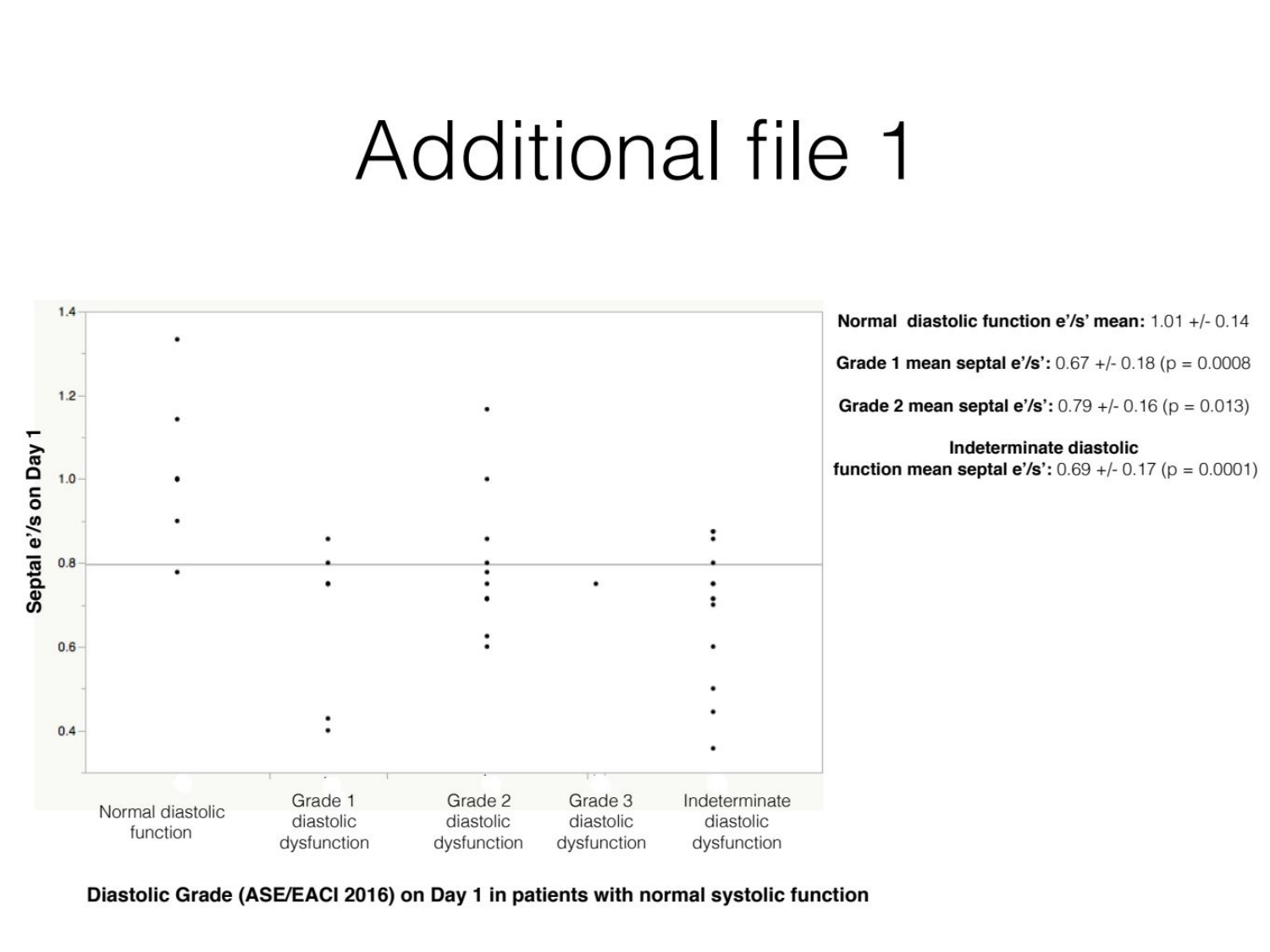

Supplement: Supplementary file 1 — Patients with normal systolic function on day 1: septal e′/s′ versus diastolic grade. (PPTX 100 kb) [file 13054_2017_1727_MOESM1_ESM.pptx]

## Slide 1
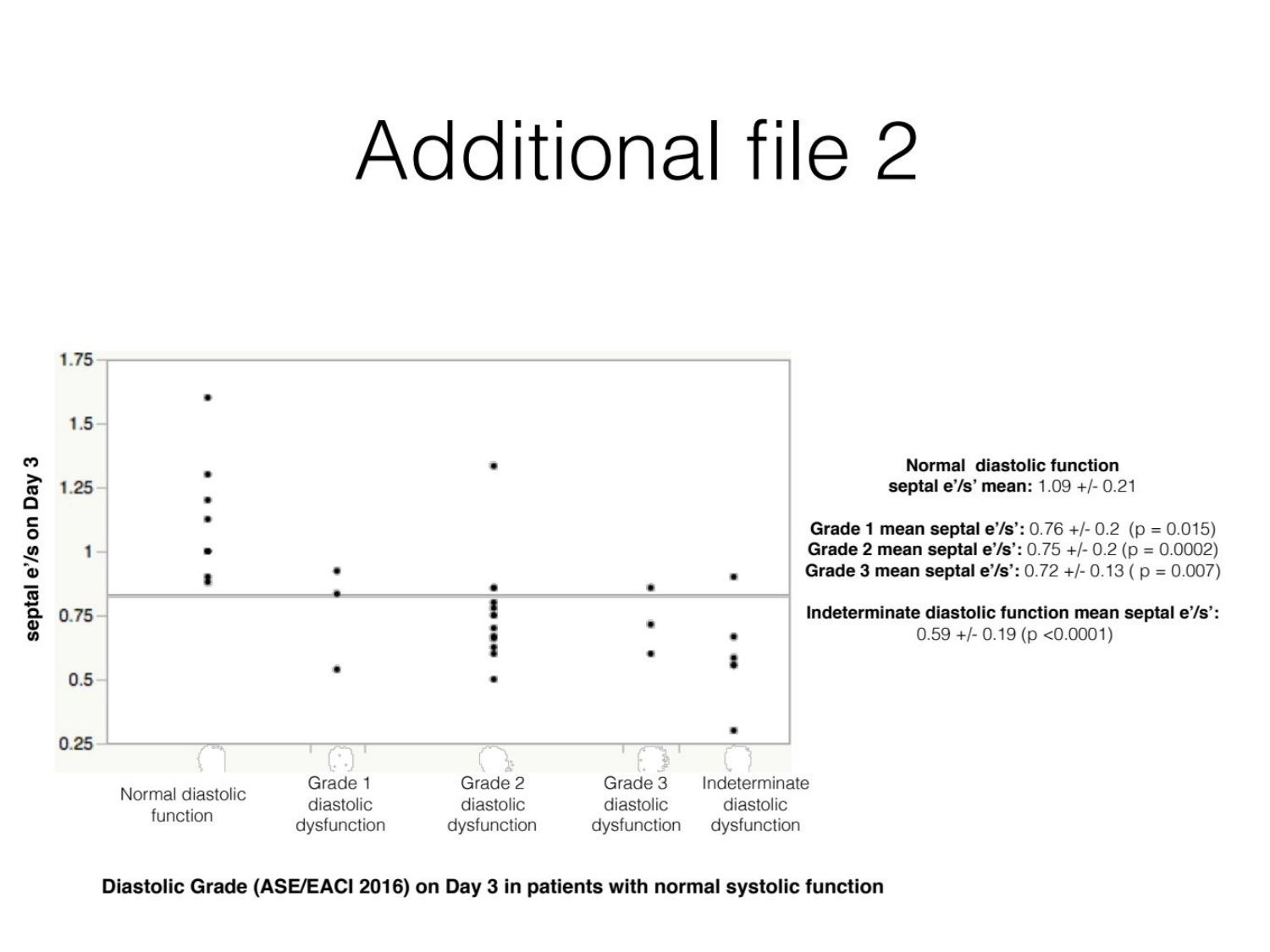

Supplement: Supplementary file 2 — Patients with normal systolic function on day 3: septal annulus e′/s′ versus diastolic grade. (PPTX 96 kb) [file 13054_2017_1727_MOESM2_ESM.pptx]

## Slide 1
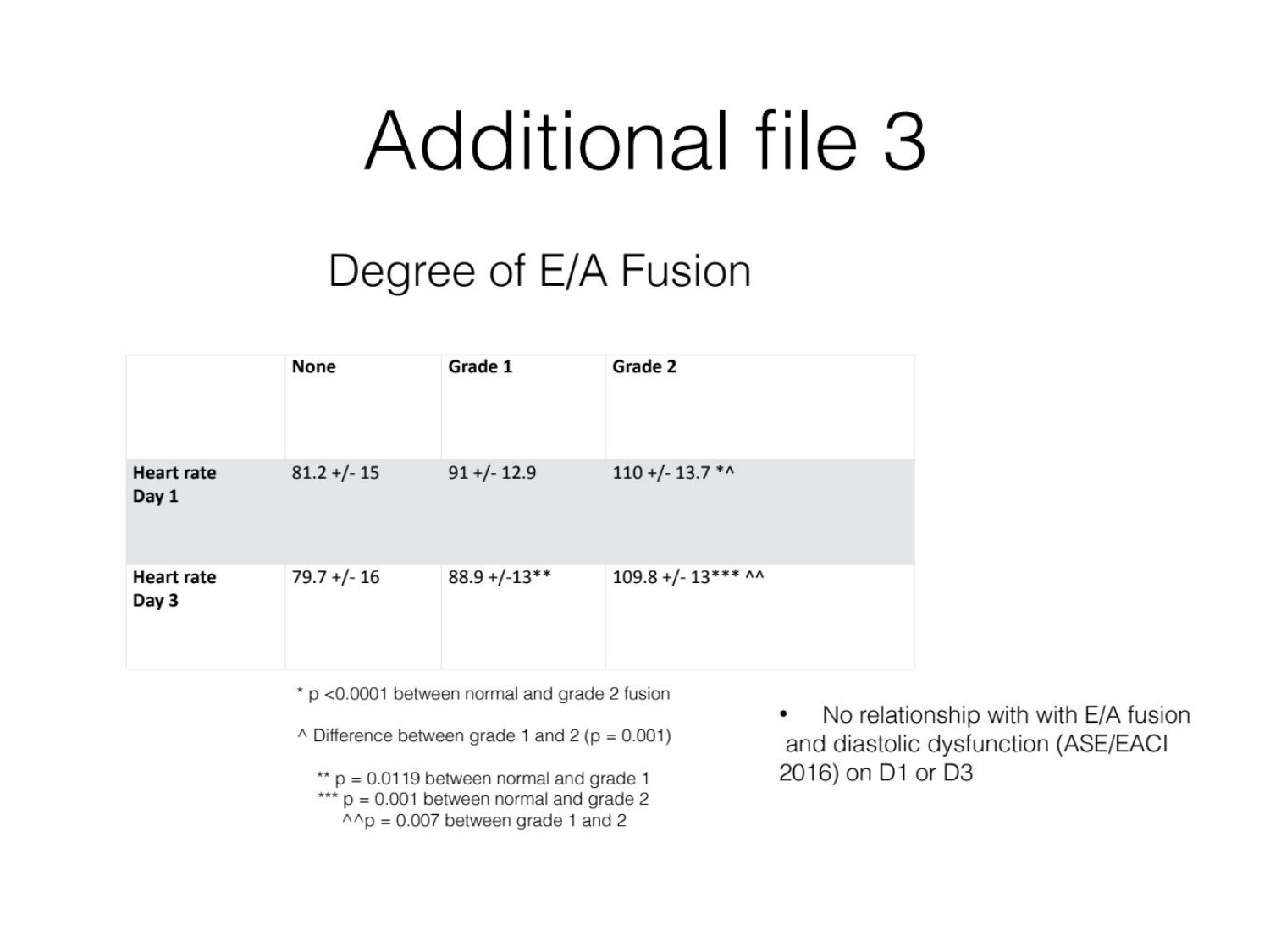

#

Supplement: Supplementary file 3 — Comparison of heart rate and grades of E/A fusion. (PPTX 184 kb) [file 13054_2017_1727_MOESM3_ESM.pptx]
